# Supplementary material for: Content-rich biological network constructed by mining PubMed abstracts
Source: BMC Bioinformatics. 2004 Oct 8;5:147. doi: 10.1186/1471-2105-5-147 (PMC528731; doi:10.1186/1471-2105-5-147)
Supplement: Additional File 2 — The original results of the above study (non-essential files are deleted to keep the file size under the limit set by BMC bioinformatics). [file 1471-2105-5-147-S2.bz2 › chilibotAdditionalFile2/dip20/648ID7922031E11035/html/CGA_CGB.html]

 


 **CGA** and **CGB** 
  
Found 505 abstracts in PubMed, retrieved 20.  
 

 What does Google say? 
 PDF only 
| .edu only 

---

**Interactive relationship** (e.g. stimulation, inhibition, etc)

**Neutral relationship**- Cross reactions of anti human chorionic gonadotropin beta  [ **CGB** ]  antibody elicited by HCG  [ **CGA** ]  beta based antifertility vaccines with other autoantigens may occur due to identical or homologous protein subsequences.  Ref: 12687122 Russ J Immunol< MedlineTA>Russ J Immunol,
- In adult islets, alpha cell granules stored  **CgA**  in the halo and  **CgB**  in both the core and the halo, beta cell granules stored both  **CgA**  and  **CgB**  in their cores, and in delta cell granules, both substances were mixed throughout the matrix.  Ref: 12826904 Pancreas, 2003

**Non-interactive relationship** (e.g. studied together, co-existance, homology, etc.)

- Strategies for diagnosing ectopic pregnancy that defer endovaginal ultrasound in women with suggestive symptoms and serum beta human chorionic gonadotropin beta  [ **CGB** ]  hCG  [ **CGA** ]  levels less than 00 mIU mL ignore the increased risk of ectopic pregnancy in these patients.  Ref: 12574008 Acad Emerg Med, 2003
- This was done to differentiate the cells into a phenotype more like syncytiotrophoblast cells as evidenced by production of beta human chorionic gonadotropin beta  [ **CGB** ]  hCG  [ **CGA** ] .  Ref: 12517328 Hypertens Pregnancy, 2002
- The unusual chromogranin B  **CgB**  chromogranin A  **CgA**  ratio with  **CgB**  in abundance over  **CgA** , previously found only in SP, was also valid for the native NP and metastasis derived prostasomes MP.  Ref: 12539232 Prostate, 2003
- Insulin was expressed in early fetal cells of both pigs and humans, and colocalization with  **CgA** ,  **CgB** , and synaptophysin was demonstrated.CONCLUSION The early expression of  **CgA**  and synaptophysin may reflect a role at early fetal stages, and the individual localization of  **CgA**  and  **CgB**  upon differentiation indicates individual functions.  Ref: 12826904 Pancreas, 2003
- CASE REPORT A 36 year old woman with stage IB 1 squamous cell carcinoma of the uterine cervix and a high serum beta human chorionic gonadotropin beta  [ **CGB** ]  hCG  [ **CGA** ]  level underwent radical hysterectomy with pelvic and para aortic lymphadenectomy.  Ref: 12477457 Gynecol Oncol, 2002
- INTRODUCTION AND AIMS To get more insight into the differentiation patterns of pancreatic islet neuroendocrine cells and granules during ontogeny, the expression and localization of chromogranin A  **CgA** , chromogranin B  **CgB** , synaptophysin, and insulin were ultrastructurally studied with the immunogold technique in porcine and human pancreatic islet neuroendocrine cells.METHODOLOGY AND RESULTS In porcine pancreas at early fetal stage,  **CgA**  was visualized throughout the immature granules in all neuroendocrine cells.  Ref: 12826904 Pancreas, 2003
- The chromogranin family includes chromogranins A  **CgA**  and B  **CgB**  and secretogranin II SgII, once called chromogranin C.  Ref: 12746559 Endocr Pathol, 2003
- With the association of some biochemical markers like maternal serum free beta human chorionic gonadotropin beta  [ **CGB** ]  hCG  [ **CGA** ]  and pregnancy associated plasma protein A PAPP A to ultrasonography at 11 14 weeks, it is possible to identify about 90% of chromosomal abnormalities.  Ref: 12635737 Clin Exp Obstet Gynecol, 2002
- The immunoreactive patterns of  **CgA**  and  **CgB**  were investigated in CJD and compared to those observed in Alzheimer s disease.  Ref: 12692477 Neuroreport, 2003
- Recent advances in chromogranin research include establishing the role of  **CgA**  in granulogenesis and the role of  **CgB**  in nuclear transcription.  Ref: 12746559 Endocr Pathol, 2003
- new biologic activities for  **CgA** ,  **CgB** , and SgII derived peptides.  Ref: 12746559 Endocr Pathol, 2003
- In the presence of placental remnants, blood was sampled for measurement of beta human chorionic gonadotropin beta  [ **CGB** ]  hCG  [ **CGA** ] , hemoglobin Hb and infectious parameters.  Ref: 12493048 Ultrasound Obstet Gynecol, 2002
- In addition to the NT, maternal serum markers as the free beta human chorionic gonadotropin beta  [ **CGB** ]  hCG  [ **CGA** ]  and pregnancy associated plasma protein PAPP A protein can improve the detection rate to 80% with a fixed 5% of FPR.  Ref: 12661334 Ginecol Obstet Mex, 2002
- Unlike  **CgA** , another granin protein  **CgB**  could not substitute for the role of  **CgA**  in secretory granule biogenesis.  Ref: 12438143 Ann N Y Acad Sci, 2002
- We found that  **CgB** , but not  **CgA** , immunoreactivity was selectively associated with prion protein deposits.  Ref: 12692477 Neuroreport, 2003
- This study was designed to evaluate the monitoring impact of urinary beta human chorionic gonadotropin beta  [ **CGB** ]  HCG  [ **CGA** ]  on tumor growth in orthotopic implantation model.  Ref: 12508539 Ai Zheng, 2002
- The patient declined surveillance with serial beta human chorionic gonadotropin beta  [ **CGB** ]  hCG  [ **CGA** ]  levels and was lost to follow up.  Ref: 12738129 Obstet Gynecol, 2003
- Primary tumors were analyzed for p53 expression using IHC staining and for beta human chorionic gonadotropin beta  [ **CGB** ]  hCG  [ **CGA** ] , hepatocyte growth factor receptor c Met, and universal melanoma antigen uMAGE messenger RNA expression using reverse transcriptase polymerase chain reaction and electrochemiluminescence.  Ref: 12470104 Arch Surg, 2002
- Diagnosis is still limited by following persistently elevated or rising postevacutation beta human chorionic gonadotropin beta  [ **CGB** ]  hCG  [ **CGA** ]  titers.  Ref: 12658908 Wien Klin Wochenschr, 2003
